# Specific Targeting of a Plasmodesmal Protein Affecting Cell-to-Cell Communication 

**DOI:** 10.1371/journal.pbio.0060007

**Published:** 2008-01-22

**Authors:** Carole L Thomas, Emmanuelle M Bayer, Christophe Ritzenthaler, Lourdes Fernandez-Calvino, Andrew J Maule

**Affiliations:** 1 John Innes Centre, Norwich Research Park, Colney, Norwich, United Kingdom; 2 Institut de Biologie Moléculaire des Plantes du CNRS (UPR2357), Strasbourg, France; 3 Université Louis Pasteur (Strasbourg 1), Strasbourg, France; Max Planck Institute for Developmental Biology, Germany

## Abstract

Plasmodesmata provide the cytoplasmic conduits for cell-to-cell communication throughout plant tissues and participate in a diverse set of non–cell-autonomous functions. Despite their central role in growth and development and defence, resolving their modus operandi remains a major challenge in plant biology. Features of protein sequences and/or structure that determine protein targeting to plasmodesmata were previously unknown. We identify here a novel family of plasmodesmata-located proteins (called PDLP1) whose members have the features of type I membrane receptor-like proteins. We focus our studies on the first identified type member (namely At5g43980, or PDLP1a) and show that, following its altered expression, it is effective in modulating cell-to-cell trafficking. PDLP1a is targeted to plasmodesmata via the secretory pathway in a Brefeldin A–sensitive and COPII-dependent manner, and resides at plasmodesmata with its C-terminus in the cytoplasmic domain and its N-terminus in the apoplast. Using a deletion analysis, we show that the single transmembrane domain (TMD) of PDLP1a contains all the information necessary for intracellular targeting of this type I membrane protein to plasmodesmata, such that the TMD can be used to target heterologous proteins to this location. These studies identify a new family of plasmodesmal proteins that affect cell-to-cell communication. They exhibit a mode of intracellular trafficking and targeting novel for plant biology and provide technological opportunities for targeting different proteins to plasmodesmata to aid in plasmodesmal characterisation.

## Author Summary

In plants, cylindrical, microscopic channels called plasmodesmata provide intracellular connections between cells for communication and material transport, and are important for many aspects of plant growth and defence. We identify a novel family of plasmodesmata-located proteins (called PDLP1) with features of type I membrane receptor-like proteins. In line with the potential for this protein to regulate molecular movement from cell to cell, we show that altered expression of the protein changes the efficiency of protein diffusion from plasmodesmata. We have also analysed the manner in which PDLP1 is transported to plasmodesmata. We show that the single transmembrane domain (TMD) of the protein contains all the information necessary for targeting to plasmodesmata and that proper targeting depends upon specific interactions with other factors within the membrane. Notably, a single amino acid close to the C-terminus of the TMD is critical for determining the intracellular destination. Further, by fusing the TMD to yellow fluorescent protein, we establish that the TMD can be used to target heterologous proteins to plasmodesmata.

## Abstract 3

Little is know about the structure and function of plant cell-to-cell connections, called plasmodesmata. This paper describes a new family of plasmodesmal proteins and the processes controlling their subcellular trafficking.

## Introduction

**Figure 1:**
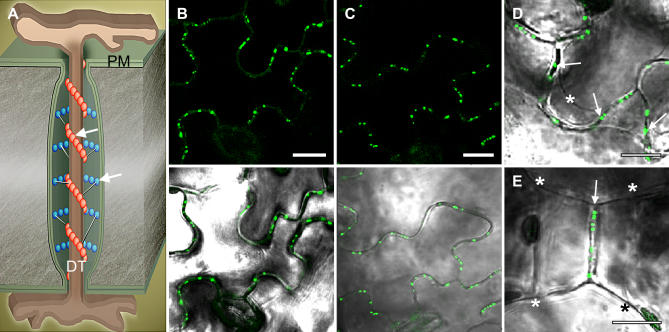
PDLP1a Targeting to Plasmodesmata (A) shows a cartoon of a plasmodesma. The plasmodesma is seen as a PM-lined channel through the cell wall containing a central ER-derived rod-shaped desmotubule (DT). The location of possible proteinaceous components (arrows), as visualised by electron microscopy [6], in the symplastic channel is illustrated. (B–D) show subcellular location of PDLP1a:GFP following transgenic expression in *Arabidopsis*. Following expression of *PDLP1a:GFP* under the control of the *CaMV 35S* promoter (B) or the *PDLP1a* native promoter (C), optical sections through the lower epidermis show the targeting of PDLP1a to plasmodesmata. The upper images show the typical punctate distribution of plasmodesmata along the cell wall of adjoining cells. The lower images show an overlay of the fluorescence on the bright field image of the same area to identify the location of the cell walls. (D and E) PDLP1a:GFP remained at punctate structures within the cell wall after plasmolysis, as shown for epidermal cells (D), and on the adjoining wall between adjacent spongy mesophyll cells (E). An overlay of the fluorescence on the bright field image of the same area is shown; asterisks (*) demarcate the retracted protoplast, and arrows identify PDLP1a:GFP retained on the cell wall. Bar indicates 10 μm.

Plasmodesmata are channels that cross the cell wall and establish symplastic continuity throughout most of the plant. Their importance has been highlighted by the range of diverse non–cell-autonomous functions that depend on intercellular macromolecular communication through plasmodesmata. Hence, a range of transcription factors in the shoot apical meristem and at the root tip have functional roles in cells other than those in which they were produced [1,2]. Similarly, some small RNAs generated as part of the RNA silencing pathway can act non–cell-autonomously [3]. Last, plant virus pathogens, which are restricted to the symplast, must use plasmodesmata to invade neighbouring cells [4], and very recently [5] it has been proposed that biotrophic fungi may exploit plasmodesmata during tissue invasion. All of these macromolecules and organisms are above the experimentally defined normal size-exclusion limits for plasmodesmata, which points to highly regulated plasmodesmal processes for their recognition and transport between cells. This is exemplified by plant viruses, all of which encode so-called movement proteins (MPs) that interact with and modify the properties of these structures to allow the passage of virus particles or other forms of ribonucleoprotein complexes [4]. The organisational complexity of plasmodesmata remains a matter of speculation constrained by the interpretation of electron microscopical images [6]. Briefly, they comprise plasma membrane (PM)-lined channels that cross the cell wall to join adjacent cells symplastically. They contain an axial-appressed membrane element derived from the endoplasmic reticulum (ER), called the desmotubule, and may contain proteinaceous spoke-like structures that cross the cytoplasmic sleeve between the PM and desmotubule (Figure 1A). Despite the crucial role of plasmodesmata in growth and development, plant defence, and pathogenesis, almost nothing is known about the integral components of plasmodesmata or plasmodesmal biogenesis. From the identification of proteins interacting with viral MPs, coimmunolocalisation studies applied to candidate proteins, and proteomics approaches, a number of proteins have been identified in association with plasmodesmata [7]. These include cytoskeletal elements (e.g., actin and myosin VIII), proteins found in the ER (e.g., calreticulin) (refs in [7]), a casein kinase 1 that phosphorylates tobacco mosaic virus (TMV) MP [8], a β-1,3-glucanase [9], and class 1 reversibly glycosylated proteins (^C1^RGPs) [10]. The lack of identification of the constituent components of plasmodesmata has remained an outstanding challenge in plant biology and has hindered a fuller understanding of the non–cell-autonomous control of plant development and the processes of tissue invasion by pathogens.

A fundamental question relating to the formation and function of plasmodesmata is how proteins are recruited to this unique subcellular environment. Previous studies have indicated the importance of components of the cytoskeleton [11–15] and the post-Golgi vesicle trafficking systems [10,16], although in no case have the specific protein sequences that define the molecular address for delivery to plasmodesmata been identified. We report here the use of Arabidopsis thaliana suspension cultures as a source of membrane proteins located in the cell wall to identify a novel family of proteins functioning within plasmodesmata to affect cell-to-cell communication, and identify the key principles by which specific subcellular targeting of these proteins is achieved.

## Results

### Plasmodesmata-Located Protein 1 Targets Plasmodesmata in Diverse Plant Species

**Figure 2:**
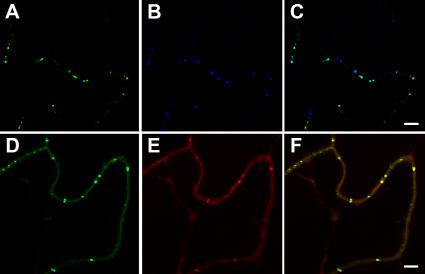
PDLP1a Resides in Plasmodesmata Following coexpression of *PDLP1a:GFP* (A and D) and *TMV MP:RFP* (E), or chemical staining of callose using aniline blue (B), colocation of PDLP1a with either marker (C and F) confirmed its localisation to plasmodesmata. Bar indicates 5 μm.

A survey of the subcellular targeting of cell wall–associated membrane proteins derived from highly purified *Arabidopsis* cell walls [17] was carried out. Translational N- and/or C-terminal fusions with green fluorescent protein (GFP) were expressed transiently using the cauliflower mosaic virus (CaMV) 35S promoter in Nicotiana benthamiana leaves and onion epidermal monolayers, and transgenically in *Arabidopsis* plants. From this survey, we identified the protein derived from *At5g43980* as a plasmodesmal protein. We refer to this newly identified protein as plasmodesmata-located protein 1a (PDLP1a). Critically, expression in transgenic *Arabidopsis* using either the CaMV 35S promoter or the native promoter for *At5g43980* showed that the fusion protein was located as punctate spots on the cell wall (Figure 1B to 1E) and that this fluorescence was retained on the wall after plasmolysis (Figure 1D and 1E). This pattern of fluorescence was most notable in leaf spongy mesophyll cells, where the punctate spots were present on adjoining walls, but absent from the nonadjoining walls (Figure 1E). Similar patterns of protein accumulation were observed following transient expression in the heterologous species N. benthamiana and onion (Figure S1). The pattern of localisation was most distinctive when PDLP1a was expressed from its own promoter, when the protein was targeted exclusively to plasmodesmata. (Figure S2A shows a confocal stack through *pPDLP1a::PDLP1a:GFP*–expressing *Arabidopsis* epidermal cells, and unique association with plasmodesmata.) Further evidence that these punctate sites were plasmodesmata was obtained by demonstrating colocalisation with callose (Figure 2A–2C) and with TMV MP:GFP (Figure 2D–2F). Callose distribution was revealed by staining with the fluorescent dye aniline blue, and was especially informative in cases where patterns of both callose and PDLP1a distribution identified the characteristic organisation of groups of plasmodesmata in pit fields (Figure S2B). TMV MP is one of the best-characterised viral MPs, which shows strong targeting to plasmodesmata in newly infected cells and in transgenic plants [4]. In transgenic plants expressing TMV MP, the protein characteristically is localised within complex plasmodesmata that are a feature of photosynthetic source tissues rather than in the simple plasmodesmata of sink tissues [18].

**Figure 3:**
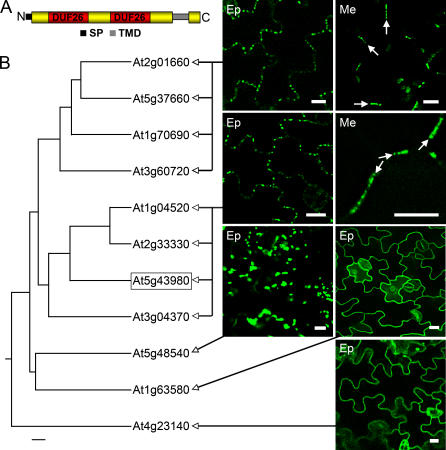
Organisation, Phylogenetic Analysis, and Subcellular Localisation of PDLP1a and Its Homologues The domain structure of PDLP1a is shown in (A). The signal peptide (SP; black box) and the TMD (grey box) were predicted using SignalP 3.0 and TMHMM 2.0, respectively. The TMD is followed by a short cytoplasmic tail. The DUF26 domain (Pfam PF01657) is shown in red. Protein homology searches using the PDLP1a amino acid sequence, followed by phylogenetic analysis (B), revealed the presence of two clades within the closely related PDLP1 family; the gene for PDLP1a is boxed. All of these genes, in addition to representatives of more distantly related DUF26 proteins, were cloned and expressed trangenically in *Arabidopsis* from the CaMV 35S promoter. Optical sections through epidermal (Ep) and spongy mesophyll (Me) tissues of transgenic *Arabidopsis* expressing representatives of the two families of *PDLP1a* homologs (*At2g33330*/clade 1 and *At2g01660*/clade 2) show punctate labelling at all areas of cell–cell contact (some are marked with arrows). Optical sections through the epidermis of transgenic *Arabidopsis* expressing a 2xDUF26 protein lacking a TMD (encoded by *At5g48540*) shows fluorescent apoplastic bodies, whereas expression of a GPI-anchored 2xDUF26 relative (encoded by *At1g63580*) shows PM labelling; bright background fluorescence from stomata is also evident. The least-related 2xDUF26 with a TMD and a C-terminal kinase domain (encoded by *At4g23140*) also shows PM labelling. Bars indicate 10 μm .

Sequence analysis showed PDLP1a to have a domain structure (Figure 3A) conserved in a small family of plant-specific proteins, including representatives from *Arabidopsis*, rice, *Medicago*, and *Phaseolus*. Briefly, the proteins are predicted to be type I membrane proteins with molecular mass ranging from 30.2 to 35.3 kDa and comprising an N-terminal signal peptide, a large region containing two similar domains annotated as domains of unknown function 26 (DUF26), a single transmembrane domain (TMD), and a short C-terminal tail. DUF26 domains have a conserved C-X8-C-X2-C motif, which is distinct from the Cys-rich regions found in S-locus glycoproteins [19]. The PDLP1 families in *Arabidopsis* and rice comprise two clades from eight members. C-terminal fusions with GFP were constructed for all eight PDLP1 proteins and analysed for their subcellular targeting after expression from the CaMV 35S promoter; all showed targeting to plasmodesmata (representative proteins from *At2g01660* and *At2g33330* are illustrated in Figure 3B). In addition, there are three other groups of DUF26-related proteins. These include a group of proteins that contain a signal peptide for secretion, but lack the TMD domain (e.g., protein from *At5g48540*, which was secreted to form large, unresolved bodies in the apoplast), a second group where the TMD and short C-terminus are replaced with a glycosylphosphoinositol (GPI) anchor domain (e.g., protein from *At1g63580*, which was localized to the PM), and a third group of predicted receptor-like kinases (e.g., protein from *At4g23140*) where the short C-terminus is replaced with a serine/threonine kinase domain. In this last case, the protein was also targeted to the PM, although in this case, the distribution was less uniform than seen for the GPI-anchored protein from *At1g63580* (Figure 3B).

### The 2xDUF26 Domain of PDLP1a Is Extracellular

**Figure 4:**
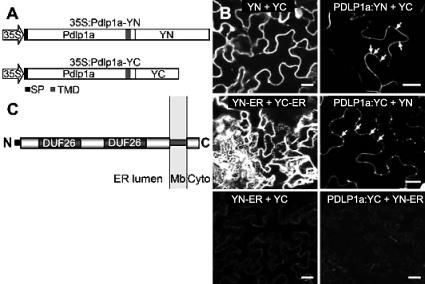
PDLP1a Membrane Topology (A) shows a schematic presentation of the expression cassettes for *PDLP1a* C-terminal fusions with *YN* and *YC* of *YFP*. N. benthamiana were agro-infiltrated with agrobacteria containing *35S::PDLP1a:YN* or *35S::PDLP1a:YC* and the corresponding counterpart (*pLH::YN*, *pLH::YC*, or *pLH::YN-ER*), and *35S::HCPro*. (B) Five days post-inoculation, fluorescence was analysed by confocal microscopy confirming YFP fluorescence reconstitution in cells coexpressing *35S::YN* and *35S::Y*C, *35S::YN-ER* and *35S::YC-ER*, *35S::PDLP1a:YN* and *35S::YC*, and *35S:PDLP1a:YC* and *35S::YN*, but not in cells coexpressing *35S::YN-ER* and *35S::YC* or *35S::PDLP1a:YC* and *35S::YN-ER*. (C) Bimolecular fluorescence confirmed the predicted orientation of PDLP1a as a type I membrane protein. Bars = 20 μm

By analogy with the wider members of the 2xDUF26 class of proteins, including some kinases that have DUF26 domains located extracellularly and signal through a TMD to the cytoplasmic kinase module [20], we predicted that PDLP1a should be orientated with its short C-terminal tail in the cytoplasm and the 2xDUF26 domain in the apoplast. To confirm this prediction, we employed a bimolecular fluorescence complementation (BiFC) strategy in which half of yellow fluorescent protein (YFP) fused to the test protein was complemented with the corresponding half of YFP targeted to the cytoplasm [21]. Hence, when either half of YFP (YN or YC, Figure 4A) was fused to the C-terminal tail of PDLP1a and cotransiently expressed in N. benthamiana with the alternate unfused half-YFP, BiFC fluorescence in plasmodesmata was observed (Figure 4B). In contrast, when PDLP1a:YC was coexpressed with the alternate unfused half carrying signals for targeting to and retention in the ER (YN-ER; [21]), no BiFC was observed (Figure 4B), confirming our predictions that PDLP1a was orientated with the C-terminus in the cytoplasm (Figure 4C). Since PDLP1 is a type I membrane protein with a single TMD, the N-terminal portion of the protein is by default located facing the apoplast.

### PDLP1 Functionality in Plasmodesmal Trafficking

The implication from the location of PDLP1 in plasmodesmata is that the protein contributes functionally to symplastic communication from cell to cell. One means of assessing this property is to measure the diffusional capacity of monomeric untargeted GFP following bombardment into single cells of leaf tissues [22] in *Arabidopsis* lines where the expression of PDLP1 genes has been changed. Wild-type *Arabidopsis* leaves cobombarded with *35S::GFP* and *35S::RFPer* (red fluorescent protein [RFP] targeted to the ER, therefore unavailable for symplastic diffusion, and used to mark target cells; Figure S3) showed the primary bombarded cells and the diffusion of free GFP to neighbouring cells where lateral diffusion could be used as a quantitative measure of plasmodesmal trafficking potential.

**Figure 5:**
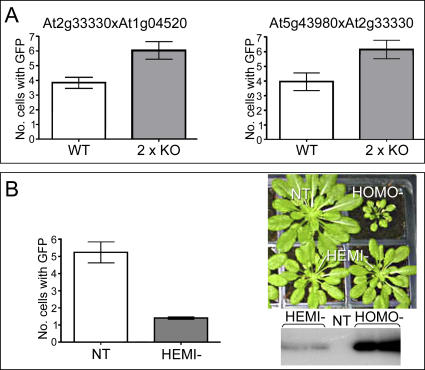
Altered Expression of *PDLP1* Changes Plasmodesmal Trafficking *PDLP1* KO lines (A) or *PDLP1a:HA* overexpressing lines (B) were assessed for plasmodesmal trafficking potential by counting the number of GFP recipient cells surrounding primary bombardment sites. From three double KO lines, only *At2g33330*×*At1g04520* and *At5g43980*×*At2g33330* showed altered GFP diffusion. (Data for *At2g33330*×*At1g04520* were obtained from two independent experiments and 96 bombardment sites each for wild-type (WT) and KO lines (*p* ≤ 0.05); data for *At5g43980*×*At2g33330* were obtained from three independent experiments and 57 and 93 bombardment sites for WT and KO lines, respectively (*p* ≤ 0.01). (B) Transgenic *Arabidopsis* plants expressing *35S::PDLP1a:HA* (or *35S::PDLP1a:GFP*; unpublished data) exhibited a dwarf phenotype (right, upper picture) when compared with nontransformed (NT) plants. This dwarf phenotype showed a positive correlation with transgene copy number and protein accumulation, assessed by western analysis with anti-HA antibody (right, lower picture). Bombardment of the less-dwarfed hemizygous plants (Hemi; left) showed a dramatic reduction in GFP diffusion (data collected from two independent experiments and 70 and 114 bombardment sites for the NT and hemizygous plants, respectively; *p* ≤ 0.0001). Homo, homozygous. Bars indicate the standard error of the mean.

Insertional mutant lines (knock-out lines; KOs) for six of the eight *PDLP1* genes are available from public collections. None of these lines showed any obvious growth or developmental phenotype. GFP diffusion in leaves of these lines was not significantly different from that in wild-type *Arabidopsis* leaves (unpublished data). However, scrutiny of the public microarray expression data (http://www.genevestigator.ethz.ch) showed that the tissue-specific pattern of expression of these genes differed widely with respect to expression in leaf tissues. To accommodate potential problems of functional redundancy within the family, various combinations of KOs were made by crossing. We concentrated on crosses between members of clade 1 (Figure 3B) that included examples of genes expressed relatively highly in leaf tissues. KO combinations of *At5g43980* and *At1g04520*, *At5g43980* and *At2g33330*, and *At2g33330* and *At1g04520* were tested in our GFP bombardment assay (Figure 5A). Whereas the homozygous progeny of *At5g43980*×*At1g04520* showed no significant change in the spread of GFP (unpublished data), the progeny of the other crosses (*At5g43980*×*At2g33330* and *At2g33330*×*At1g04520*) showed significantly increased trafficking ability (*p* ≤ 0.05 and ≤ 0.01, respectively).

The corollary to increased trafficking with reduced PDLP1 was tested by assessing GFP spread in transgenic plants overexpressing *PDLP1a* from the CaMV 35S promoter. Transgenic plants expressing genes for either *PDLP1a:GFP* or haemaglutinin-tagged *PDLP1a* (*PDLP1a:HA*) showed a reduced-growth phenotype that correlated with transgene copy number, i.e., plants with homozygous single insertions were more dwarfed than heterozygous plants (Figure 5B) and this dwarfing phenotype positively correlated with protein accumulation, assessed using anti-HA antibody (Figure 5B). To minimise the contributory effect of the dwarf phenotype and to avoid the complication of transgene silencing of GFP after bombardment, GFP diffusion assays were carried out on heterozygous plants of the *PDLP1a:HA* line. Measurements of lateral diffusion from sites of bombardment in these plants showed that cell-to-cell trafficking of GFP was highly significantly impaired (*p* ≤ 0.0001; Figure 5B).

### PDLP1a Utilises the Secretory Pathway for Delivery to Plasmodesmata

**Figure 6:**
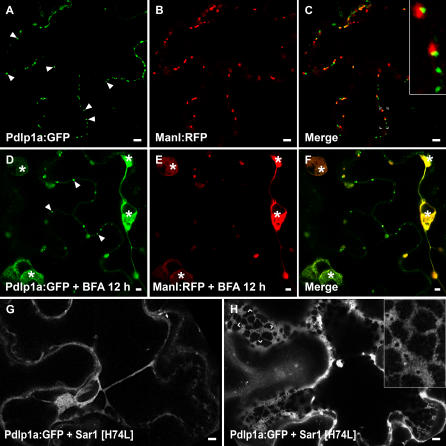
PDLP1a Utilises the Secretory Pathway for Delivery to Plasmodesmata N. benthamiana leaves were agro-infiltrated with a construct expressing *35S::PDLP1a:GFP* (A to H) and either a construct expressing a Golgi marker (*35S::ManI:RFP*; [A to F]) or a construct expressing the Sar1 inhibitory mutant variant Sar1[H74L]:RFP that blocks ER to Golgi transport (G and H). (A–C) Cells expressing *35S::PDLP1a:GFP* and *35S::ManI:RFP* showed discrete punctate structures characteristic of plasmodesmata (arrowheads), and Golgi stacks, respectively. Inset, high magnification of the boxed region in (C) confirms the discrete nature of the two subcellular locations. (D–F) Treatment with BFA led to a severe reduction in number of cell wall–associated PDLP1a:GFP-labeled punctate structures ([D], compare to [A], arrowheads), and the near complete loss of individual Golgi stacks ([B], compare to [E]), and concomitantly in the formation of typical BFA compartments (asterisks) in which both PDLP1a:GFP and ManI:RFP colocalized. (G and H) Similar phenomenon was observed upon coexpression of *PDLP1a:GFP* and the GTP-restricted mutant *Sar1[H74L]*. In median section (G), PDLP1a:GFP formed large aggregates in the nuclear vicinity that resembled the BFA compartment. Fluorescence extended also all around the cells and within cytoplasmic strands (G). In the cortex of the same cells (H), PDLP1a:GFP was found to be located in a typical ER polygonal network that was better seen upon higher magnification (inset, 3× magnification of the boxed region in [H]). Coexpression of the *Sar1[H74L]* and *PDLP1a* constructs in the same cell was confirmed from their respective fluorescent tags. Scale bars indicate 5 μm.

Trafficking of proteins to the plasmodesmata has variously been shown to exploit (e.g., ^C1^RGP [10]) or bypass (e.g., TMV MP; [23,24]) the secretory pathway. Chemical or protein inhibitors were used to investigate the role of the secretory pathway in PDLP1a trafficking to plasmodesmata. Brefeldin A (BFA), an inhibitor of specific ADP ribosylation factor (ARF) GTPase exchange factors (GEFs), arrests vesicle trafficking at various points along the secretory pathway [25], whereas Sar1[H74L], a GTPase-defective mutant of Sar1p, very specifically affects COPII-mediated ER-to-Golgi transport [26]. Both of these inhibitors provided a qualitative assessment of the roles of the ER and COPII pathways in PDLP1a targeting to plasmodesmata. Coexpression of *PDLP1a:GFP* and *ManI:RFP*, encoding a well-described secretory marker whose translocation to the Golgi apparatus depends on a functional secretory pathway [27], resulted in the identification of PDLP1a:GFP at plasmodesmata, and distinct and separate Golgi labelling with ManI:RFP (Figure 6A to 6C). Addition of BFA led to the complete loss of Golgi stacks and the concomitant formation of large ER–Golgi hybrid bodies/BFA compartment in which both PDLP1a:GFP and ManI:RFP accumulated (Figure 6D to 6F, stars). In addition to this location, PDLP1a:GFP could still occasionally be seen within plasmodesmata after treatment (compare Figure 6A and 6D, arrowheads). This was likely due to a (dose-dependent) incomplete inhibition of secretion or to pre-existing PDLP1a:GFP molecules within plasmodesmata before addition of BFA. Since BFA induces a range of effects on treated cells due to the inhibition of multiple Arf-GEFs [25], additional experiments were performed using a dominant-negative mutant of the Ras-like small GTPase, Sar1 (*Sar1[H74L]*), to specifically block ER export; the wild-type protein was used as the control. As expected, coexpression of wild-type *Sar1:RFP* with *PDLP1a:GFP* had no effect on PDLP1a:GFP steady-state accumulation within plasmodesmata (unpublished data). In contrast, coexpression with the GTPase-impaired mutant *Sar1[H74L]* led to the retention of PDLP1a:GFP in the ER as deduced from the perinuclear labelling (Figure 6G) and the visualisation of a typical polygonal network in cortical sections (Figure 6H). Similar to BFA treatment, incorporation of PDLP1a into plasmodesmata was not completely abolished in all cells. This could be as a consequence of partial inhibition of secretion following expression of *Sar1[H74L]*, because inhibition of secretion is directly related to the steady-state accumulation of Sar1[H74L] [28], or prior accumulation of PDLP1a:GFP before *Sar1[H74L]* expression. Such variability is inherent in experiments using transient expression following agro-infiltration. Most importantly, however, induced retention of PDLP1a:GFP in the ER or in the BFA compartment was never observed under conditions where secretion was unperturbed. It is therefore concluded that PDLP1a utilises the secretory pathway for delivery to plasmodesmata.

### PDLP1 TMD Is Necessary and Sufficient for Plasmodesmal Targeting

**Figure 7:**
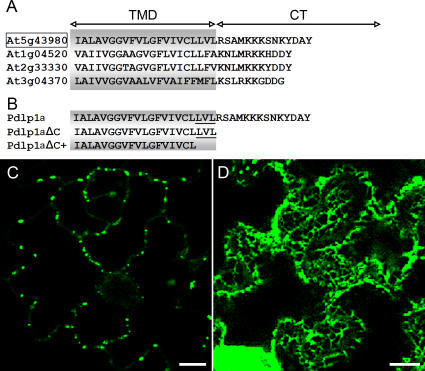
Mutation Analysis of the Transmembrane Domain and Cytoplasmic Tail of PDLP1a (A and B) Multiple sequence alignment showing homology in the TMD within clade 1 of the PDLP1 family and greater variation in the cytoplasmic tail (CT) (A); the gene for PDLP1a is boxed. Deletions were made in the TMD and cytoplasmic tail (CT) of PDLP1a (B). (C) After transgenic expression in *Arabidopsis*, PDLP1aΔC:GFP deleted for the CT targeted to punctate structures in the cell wall. (D) In contrast, transgenic expression to produce PDLP1aΔC+:GFP deleted for the CT plus the three amino acids (LVL; underlined) at the end of the TMD, abolished punctate labelling in the cell wall and resulted in targeting to the ER. Bars indicate 10 μm.

Bioinformatic analysis of the PDLP1 family clade 1 identified a common TMD of 21 amino acids upstream of a short but variable length C-terminal tail (Figure 7A). To assess the importance of the C-terminal tail in directing PDLP1a to plasmodesmata, this region was deleted (Figure 7B) and the resulting C-terminal fusion to GFP tested for targeting to plasmodesmata. Transient and transgenic expression showed that the protein lacking the C-terminal tail was still targeted to plasmodesmata (Figure 7C).

**Figure 8:**
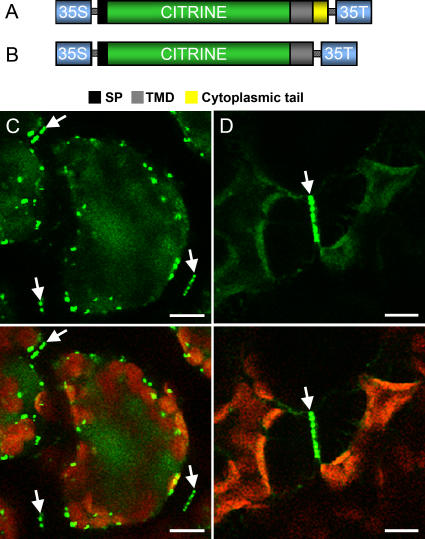
The TMD of PDLP1a Is Sufficient to Target Foreign Proteins to Plasmodesmata (A and B) Chimeric constructs consisting of the signal peptide from PDLP1a, followed by the coding sequence for YFP (citrine) fused to the TMD and CT of PDLP1a (A), or the TMD alone (B), were constructed and expressed. (C and D) Upon plasmolysis of spongy mesophyll cells, the chimeric protein remained in the cell wall at the areas of cell–cell contact for both YFP:TMD+CT (C) and YFP:TMD (D) proteins. The former also showed accumulation of fluorescent bodies within the cytoplasm. Overlays of the YFP fluorescence and red chloroplast autofluorescence (bottom) images clearly show YFP labelling retained after plasmolysis at the points of cell-to-cell contact (arrows). Bars indicate 10 μm.

That PDLP1a was targeted to plasmodesmata in the absence of the C-terminal tail led us to ask whether the TMD alone would be sufficient. To assess this, two constructs were tested. The citrine variant of YFP (this has higher stability in the acidic extracellular environment) [29] was fused between the PDLP1a N-terminal signal peptide and the TMD plus C-terminal tail, or the TMD alone (Figure 8A and 8B). In both cases, the YFP open reading frame was flanked with flexible linker peptides [30], minimizing protein folding interference between the YFP and PDLP1a domains. Transgenic or transient expression of the latter fusion protein showed unique targeting to plasmodesmata, which was unaffected by plasmolysis (Figure 8C and 8D, arrows). For the former construct including the C-terminal tail, similar targeting was observed except that YFP fluorescence was also visible at discrete unidentified locations in the cytoplasm (Figure 7C). Therefore, our experiments have uniquely identified a short TMD sequence with the capacity to target proteins to plasmodesmata.

The specification of plasmodesmal targeting by the TMD alone identifies either a default pathway based upon some physical property of the TMD (e.g., TMD length), or that the TMD includes specific targeting signals. To test these hypotheses, we focussed on the C-terminal three amino acids of the TMD. It has been proposed [31] that a TMD-length rule applies to some proteins translocated along the secretory pathway to correspond with the increasing thickness of the membranes along the path. Hence, frequently, ER proteins have shorter TMDs than do PM proteins [31,32]. In agreement with this model, a construct with a further deletion of three amino acids at the C-terminus of the TMD, shortening it to 18 amino acids (Figure 7B), was no longer targeted to plasmodesmata, but was retained within the ER (Figure 7D).

The three C-terminal amino acids in the PDLP1a TMD comprise the hydrophobic amino acids LVL. Alanine-substitution mutants were made in which individual or combinations of amino acids were replaced with alanine to test whether these three amino acids, in part, specified plasmodesmal targeting; alanine substitution preserved the length of the hydrophobic TMD. Single or combined mutations revealed only two targeting phenotypes. Changes that flanked the central V residue continued to be targeted to plasmodesmata. Any changes that included the central V→ A change resulted in retention of PDLP1a in the ER (Table 1).

**Table 1:**
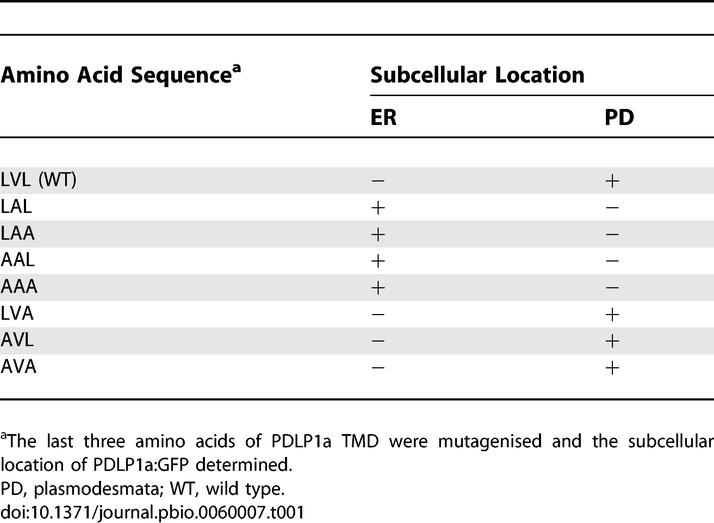
Targeting of PDLP1a to Plasmodesmata Can Be Determined by a Single Amino Acid in the TMD

## Discussion

Until now, progress on the identification of the protein constituents of plasmodesmata has been remarkably slow. Being embedded within the relatively rigid cell wall structure has hindered plasmodesmal purification using conventional biochemical approaches and delayed progress on understanding these molecular channels that lie at the heart of cellular communication in plants. Our approach, based upon a bioinformatic analysis of a partial *Arabidopsis* cell wall proteome [17], has identified PDLP1 proteins as a family of novel plasmodesmal constituents. Other plasmodesmata-associated proteins have been identified (cited earlier), but in the majority of cases, these appear to have multiple subcellular locations that could reflect various cellular roles. By drawing parallels with the nuclear complex, which could be considered to have analogous functions in the translocation of macromolecules, plasmodesmata should have many tens of proteins involved in their intrinsic structure and function and which may be uniquely located in that environment. PDLP1a expressed from its own promoter appears to uniquely accumulate in plasmodesmata and, hence, *pPDLP1a::Pldp1a:GFP* transgenic plants provide a valuable marker line for the location and frequency of plasmodesmata. In contrast to TMV MP, which only accumulates in complex plasmodesmata that are a feature of photosynthetic source tissues, PDLP1a:GFP accumulates more widely. It was identified from rapidly dividing suspension cells that contain only simple primary plasmodesmata [33] and identifies plasmodesmata in both very young (photosynthetic sink; unpublished data) and mature leaf tissues.

The PDLP1 family resides within a larger group of proteins that share the 2xDUF26 domain configuration in common. Some of these have features of receptor-like kinases that are induced in response to treatments with pathogens or signalling molecules associated with pathogen attack (e.g., reactive oxygen species and salicylic acid [20,34,35]). These kinases have DUF26 domains located extracellularly and signal through a TMD to the cytoplasmic kinase module [20]. In accord with the predictions resulting from bioinformatic analysis suggesting that PDLP1a is a type I membrane protein, we showed that the short C-terminal tail of PDLP1a resides within the cytoplasm, leaving the 2xDUF26 domain in the apoplast. It is intriguing that such receptor-like molecules at the PM should be uniquely targeted to plasmodesmata and suggests that extracellular signals could be transduced directly into the environs of the plasmodesma to control molecular trafficking between cells.

That PDLP1 proteins have the potential to modulate cell-to-cell trafficking was clearly established by altering their expression and measuring the effect in a GFP diffusion assay. This assay has been used widely as a measure of plasmodesmal function [22]. Lines overexpressing *PDLP1a* showed a dramatic reduction in GFP movement. Interestingly, the homozygous transgenic lines showing a large ectopic accumulation of PDLP1 also showed a reduced-growth phenotype indicative of the damaging effect of reduced cell-to-cell communication. The logical corollary of the reduced trafficking would be increased trafficking when PDLP1 proteins were reduced in their expression. However, this makes a number of assumptions: First, that in normally growing plants, the PDLP1 protein is constitutively active, and second, that the effects on single genes would not be masked due to redundancy with other members of the multigene family. The diverse expression patterns of the PDLP1 proteins are distinctive but show extensive overlap across different tissues. Hence, we were not surprised to find that single insertional KO lines showed no visible phenotype and no change in GFP movement. However, by concentrating on clade 1 of the PDLP1 family, within which there are members expressed relatively highly in leaves (the test tissues for our GFP assay), we showed that combined KO lines did exhibit the reverse phenotype (i.e., more GFP movement). Three double KO combinations were tested, but only two showed increased GFP trafficking. We assume that the effect of these combined mutations is dependent upon the relative contribution each gene makes in wild-type plants and that extending the combinations further (e.g., triple and quadruple KOs) would increase the likelihood of an altered response and, possibly, ultimately some negative impact upon plant performance. Uncovering the contribution of the PDLP1 proteins to plasmodesmal function will add important information to our very limited understanding of plasmodesmal control.

Little has been known of the mechanisms, the chaperones, or the addresses that specify plasmodesmal targeting. For the three best-characterised plasmodesmal-associated proteins, TMV MP, β-1,3-glucanase, and the ^C1^RGPs, the intracellular route to plasmodesmata is different. TMV MP is a membrane-embedded protein [36], but in its targeting to plasmodesmata, is BFA-insensitive [24] and likely involves the actin cytoskeleton [24]. In contrast, targeting of the ^C1^RGPs, which are soluble and not membrane proteins [37], depends on active secretion [10] although they lack an N-terminal signal peptide. The RGPs have no established function, and the determinants for plasmodesmal targeting remain to be identified. The plasmodesmal β-1,3-glucanase is a GPI-anchored protein [9] that is presumably secreted. It is intriguing that this protein partitions between a general PM location and plasmodesmata, but is not retained at the latter site after plasmolysis [9]. This implies that unlike PDLP1a, the β-1,3-glucanase moves to plasmodesmata via the PM and sustains only a superficial physical association with plasmodesmata.

The PDLP1 family of proteins have structural features typical of secreted proteins and are targeted to plasmodesmata via the secretory pathway. Hence, PDLP1a accumulates in the BFA compartment after treatment and shows sensitivity to disruption of the COPII-mediated ER-export by the GTP-locked form of Sar1. In addition, it is well established that the destination along the secretory pathway for single-pass membrane proteins is influenced markedly by the length of the hydrophobic domains [31,32]. Thus, lengthening the TMD of the plant vacuolar sorting receptor BP-80 from 19 to 22 amino acids resulted in the escape of the protein from the Golgi and its accumulation in the PM [31]. Conversely, reducing the length of the TMD of another type I protein from 23 to 20 or to 17 residues led to the retention of the protein in the Golgi or the ER, respectively [31]. The same rule is likely also to apply to PDLP1a and other members of the family. With its predicted 21 amino acids, the TMD is probably sufficient to promote exit from the ER and the trafficking of the protein to the Golgi or even to the PM, a hypothesis further substantiated by the ER retention of the PDLP1aΔC^+^:GFP mutant with a shortened TMD. We exclude a contribution from the PDLP1a signal peptide since across the eight members of the family, the signal peptides show no amino acid homology except that they fulfil the hydrophobic and approximate length requirements of signal peptides. It seems very unlikely, however, that the length of the TMD on its own would be sufficient to specify plasmodesmal targeting. Rather, our results where we have mutated the TMD while retaining its predicted length of 21 amino acids strongly support the presence of a sorting signal that would be recognized by a receptor system and machinery (specific vesicles; intermediate compartments; and trafficking regulatory proteins) for delivery to the correct destination. Sorting signals of intrinsic membrane proteins generally reside within the extramembranous domains (exposed toward the cytoplasm or the ER lumen) [38,39]. In the absence of such exposed determinants, as demonstrated by plasmodesmal targeting of the chimeric SP:Citrine:TMD (Figure 8) that completely lack the N- and C-terminal extramembranous domains of PDLP1a, such a sorting signal must reside within the TMD and its recognition must take place within the lipid bilayer. The most likely scenario would be for this to take place through lateral interaction with other TM proteins. Further experiments are needed to address the existence of such sorting signal mechanisms for the targeting of PDLP1-type and other proteins to plasmodesmata. Also it will be important to determine whether sorting occurs during secretion, i.e., within the Golgi complex or trans-Golgi network where most proteins are sorted, or only once PDLP1a has reached the PM.

Although we understand little at present about the structure and function of plasmodesmata, that PDLP1a TMD is sufficient to confer plasmodesmal targeting opens up the possibility of targeting other novel functions to this unique subcellular compartment with the potential to selectively control the passage of macromolecules or for the more general control of molecular trafficking.

## Materials and Methods

### Cloning.

Gateway technology (Invitrogen) was used to generate all the clones in this publication. The primer sequences used for cloning and mutagenesis are available upon request. Gene sequences were amplified by PCR using Phusion DNA polymerase (NEB). The resulting DNA fragments were purified and transferred by recombination into the entry vector pDONR207 (Invitrogen) using BP clonase II (Invitrogen) following the manufacturer's conditions. The sequence of the resulting pDONR clone was verified by automated sequencing. The *PDLP1a* sequence was transferred by recombination to the indicated binary destination vector using LR clonase II (Invitrogen) following the manufacturer's conditions.

*PDLP1* coding sequences (CDS) were amplified from a pool of cDNA made from Arabidopsis thaliana Col-0 RNA and recombined into *pDONR207* before transfer by recombination to the binary destination vector *pB7FW2.0* [40] to give *35S::PDLP1:GFP*. Subsequent recombination into pEarleygate301 [41] generated *35S::PDLPa.HA*. *PDLP1a* regulatory sequences were amplified from 1.5 kbp upstream of the ATG of *PDLP1a* (promoter) and to the end of the CDS. *PDLP1a* and *eGFP* were combined using overlap PCR with attB adaptor primers and was recombined into *pDONR207* and then to the binary destination vector *pEarleygate301* to give *pPDLP1a::PDLP1a:GFP*.

PCR mutagenesis of *PDLP1a* was used to create deletions of the TMD and cytoplasmic tail. All cloning was again carried out using Gateway technology with the entry vector *pDONR207* and destination vector *pB7FWG2.0*.

Genes for GPI-anchor proteins specify N- and C-terminal targeting signals, which are cleaved to generate the mature protein. Hence, *At1g63580* was cloned with a modified [42] citrine variant of *YFP* [29] inserted as a translational fusion between the N-terminal secretion signal and the CDS using overlap PCR. Similar constructs were made to fuse PDLP1a N-terminal secretion signal to YFP and the TMD or TMD+C-terminal of PDLP1a.

For BiFC, overlap PCR was used to fuse the CDSs for two nonfluorescent *YFP* fragments, *YFP_1–154_ (YN)* and *YFP_155–239_ (YC)* from *pRT-YN* and *pRT-YC* [21] to the C-terminus of the *PDLP1*a CDS, giving *35S::PDLP1a:YN* and *35S::PDLP1a:YC*.

Reporter constructs for bombardment were *eGFP* and *RFPe*r, cloned into *pK7WG2* [40]. For the latter, overlap PCR was used to add the secretion signal sequence from Bip2 (*At5g42040*) and the KDEL ER-anchor sequence to the N- and C- termini, respectively.

### Bombardment experiments.

Particle bombardment followed published methods [43,44]. A total of 5 μg of each DNA plasmid was mixed and precipitated onto 1-μm gold particles (Bio-Rad). Fully expanded *Arabidopsis* leaves on plates (MS medium + 0.8% agar) were bombarded twice. GFP diffusion, counted as the numbers of secondary cells surrounding the primary target site (shown by RFPer), was analysed 24 h post-bombardment by confocal microscopy. Statistical nonparametric Mann-Whitney analysis was performed using Graph Prism software (GraphPad Software).

### In vivo expression.

Binary clones were transformed into Agrobacterium tumefaciens GV3101 using electroporation, and infiltrated into leaf tissue under gentle pressure using a syringe barrel, or used for plant transformation. For leaf infiltration, bacteria were grown overnight in LB plus the appropriate antibiotics, collected, and then resuspended in 3 ml of 10 mM MgCl2 containing 100 μM acetosyringone. After a minimum of 2 h at room temperature, the culture was diluted to an optical density at 600 nm (OD_600_) of 0.2–0.5.

*Arabidopsis* flower dip transformation was carried out according to [45]. Transgenic seedlings were identified by spraying the germinated T0 seedlings with Challenge (Bialophos herbicide) and screening the T0 survivors using a confocal microscope.

### Transient expression in N. benthaniana.

Two leaves on a young plant at the four-leaf stage were pressure infiltrated with the bacterial culture and left for 48–60 h before visualising using the confocal microscope. For the membrane topology experiment, bacteria carrying *pLH::YN*, *pLH::YC*, *pLH::YNER* (gifts from A. Zamyatnin), *35S::PDLP1a:YN*, *35S::PDLP1a:YC*, or *pBIN::HcPro* (a viral silencing suppressor included to enhance expression of the -YN and -YC fusions [21]) were mixed in the appropriate -YC and -YN combinations together with *pBIN::HcPro*, and infiltrated into N. benthamiana leaves. Leaves were analyzed at 5 d post-infiltration by confocal microscopy. (The *35S::PDLP1a:YN*/*pLH::YCER* combination has been excluded from the reported data since, as indicated in Zamyatnin et al., [21], it gave rise to high background fluorescence; unpublished data).

### Transient expression in onion monolayers.

The epidermis was dissected from the onion and placed on plates containing MS medium + 6% agar. Biolistic bombardment using the BioRad gene gun delivered gold particles coated in *35S::PDLP1a:GFP*. The onion epidermis was incubated in a growth room for 48–60 h before being visualised using a confocal microscope.

### Plasmolysis.

Transformed plant tissue was infiltrated with 30% glycerol and viewed immediately.

### Staining of callose with aniline blue fluorochrome.

Aniline blue fluorochrome (Biosupplies**)** was used at 0.1 mg/ml and infiltrated into the transformed plant tissue before being analysed by confocal microscopy.

### Confocal microscopy.

Plant tissue was imaged at room temperature using a Leica TCS SP2 inverted confocal microscope with an Argon ion laser. GFP was excited at 488 nm and the emitted light captured at 505 to 555 nm; light emitted at 630–680 nm recorded chlorophyll autofluorescence. YFP was excited at 514 nm, and the emitted light was captured at 525–650 nm. RFP was excited using 543 nm and captured at 590–630 nm. Aniline blue fluorochrome was excited with a 405-nm laser (30% strength) and the emitted light captured between 460–500 nm. Images were captured digitally and handled using the Leica LCS software. Alternatively (Figure 6; inhibitor experiments), protein location was similarly assessed using a Zeiss LSM510 confocal microscope with equivalent settings.

### Inhibitor studies.

Inhibitor studies were carried out following transient expression of *35S::PDLP1a:GFP* in 4-wk-old N. benthamiana plants. To differentiate the location of *35S::PDLP1a:GFP* from Golgi, plants were coinfiltrated with a construct expressing *ManI:TdTomato*, named *ManI:RFP* throughout the text. To implicate the secretory pathway in PDLP1a targeting to plasmodesmata, leaves were infiltrated with BFA (50 μg/ml in water) 40 h after infiltration with the expression constructs and examined after a further 12 h. Water controls were run in parallel. To determine the role of COPII-mediated ER-export in PDLP1a targeting, *35S::PDLP1a:GFP* was coinfiltrated with *35S::Sar1:RFP* or the mutant *35S::Sar1[H74L]:RFP*. To create *35S::Sar1[H74L]*, *NtSar1* [46] was first cloned into *pDONR Zeo* (Invitrogen) using GATEWAY cloning to generate *pDONR Sar1* that was used as a template for site-directed mutagenesis. In-frame C-terminal fusions between wild-type or mutated Sar1 and mRFP were generated by recombination using *pH7RWG2* vector [40].

### Bioinformatics.

Web-based bioinformatic tools were used in the identification and analysis of PDLP1 homologues. The SMART database (http://smart.embl-heidelberg.de/) was used to identify proteins with a similar domain structure to PDLP1a, and their similarity compared using BLAST. The homologs were aligned using Clustal (http://www.ebi.ac.uk/clustalw/), and the alignment output from Clustal was put into Treetop Phylogenetic tree prediction (http://www.genebee.msu.su/services/phtree_reduced.html) using the Phylip format. The distances that came out of this prediction were put into Phylodendron phylogenetic tree printer (http://iubio.bio.indiana.edu/treeapp/treeprint-sample1.html) to draw the phylogenetic tree.

## Supporting Information

Figure S1 PDLP1a:GFP Targeting in Species Other Than *Arabidopsis*
Optical sections through the epidermis of N. benthamiana transiently expressing *35S::PDLP1:GFP* show PDLP1:GFP targeted to punctate spots on the cell wall (A) and to the cell wall interface at the base of the leaf trichome (arrow [B]), a region with abundant plasmodesmata. Onion epidermal strips were bombarded with *35S::PDLP1:GFP*-coated particles. After 60-h incubation, punctate spots were clearly visible on the walls at the interfaces between adjacent cells (C). The lower panels of (A) and (C) show overlays of the fluorescence on the bright field image of the same area, that of (B) shows just a bright field image of the same area to visualise the base of the trichome (arrow). Bars indicate 10 μm.
(8.5 MB TIF)

Figure S2 Subcellular Localisation of PDLP1a:GFP (A) shows a confocal stack of GFP localisation following expression of *pPDLP1a::PDLP1a:GFP* in transgenic *Arabidopsis* and reveals punctate labelling only associated with plasmodesmata. (B) shows the accumulation of PDLP1a:GFP at pit fields containing clusters of plasmodesmata. Pit fields can be seen from the side (boxed area 1) or in front views (boxed area 2). Right panels represent 4× magnification of boxed areas 1 and 2. Bar indicates 10 μm in (A) and 5 μm in (B).
(2.2 MB TIF)

Figure S3 Free GFP Diffusion as a Plasmodesmal Trafficking Assay Arabidopsis leaves bombarded with gold particles coated with *35S::GFP* and *35S::RFP*er plasmids show the restriction of RFPer to the target cell and diffusion of the free GFP to surrounding cells. Bar indicates 33 μm.
(4.2 MB TIF)

## Abbreviations

BFA: Brefeldin A
BiFC: bimolecular fluorescence complementation
CaMV: Cauliflower mosaic virus
DUF: domain of unknown function
ER: endoplasmic reticulum
GFP: green fluorescent protein
GPI: glycosylphosphoinositol
KO: knock-out line
MP: viral movement protein
PM: plasma membrane
PDLP1: plasmodesmata located protein 1
RFP: red fluorescent protein
RGP: reversibly glycosylated protein
TMV: tobacco mosaic virus
TMV MP: tobacco mosaic virus movement protein
TMD: transmembrane domain
YC: C-terminal half of yellow fluorescent protein
YFP: yellow fluorescent protein. YN, N-terminal half of yellow fluorescent protein.

## References

[pbio-0060007-b001] Kurata T, Okada K, Wada T (2005). Intercellular movement of transcription factors. Curr Opin Plant Biol.

[pbio-0060007-b002] Lee J-Y, Colinas J, Wang JY, Mace D, Ohler U (2006). Transcriptional and posttranscriptional regulation of transcription factor expression in Arabidopsis roots. Proc Natl Acad Sci U S A.

[pbio-0060007-b003] Dunoyer P, Himber C, Voinnet O (2005). DICER-LIKE 4 is required for RNA interference and produces the 21-nucleotide small interfering RNA component of the plant cell-to-cell silencing signal. Nat Genet.

[pbio-0060007-b004] Lucas WJ (2006). Plant viral movement proteins: agents for cell-to-cell trafficking of viral genomes. Virology.

[pbio-0060007-b005] Kankanala P, Czymmek K, Valent B (2007). Roles for rice membrane dynamics and plasmodesmata during biotrophic invasion by the blast fungus. Plant Cell.

[pbio-0060007-b006] Ding B, Turgeon R, Parthasarathy MV (1992). Substructure of freeze-substituted plasmodesmata. Protoplasma.

[pbio-0060007-b007] Oparka KJ (2004). Getting the message across: how do plant cells exchange macromolecular complexes?. Trend Plant Sci.

[pbio-0060007-b008] Lee J-Y, Taoka K-I, Yoo B-C, Ben-Nissan G, Kim D-J (2005). Plasmodesmal-associated protein kinase in Tobacco and Arabidopsis recognizes a subset of non-cell-autonomous proteins. Plant Cell.

[pbio-0060007-b009] Levy A, Erlanger M, Rosenthal M, Epel B (2007). A plasmodesmata-associated β-1,3-glucanase in Arabidopsis. Plant J.

[pbio-0060007-b010] Sagi G, Katz A, Guenoune-Gelbart D, Epel BL (2005). Class 1 reversibly glycosylated polypeptides are plasmodesmal-associated proteins delivered to plasmodesmata via the Golgi apparatus. Plant Cell.

[pbio-0060007-b011] Gillespie T, Boevink P, Haupt S, Roberts AG, Toth R (2002). Functional analysis of a DNA-shuffled movement protein reveals that microtubules are dispensable for the cell-to-cell movement of tobacco mosaic virus. Plant Cell.

[pbio-0060007-b012] von Bargen S, Salchert K, Paape M, Piechulla B, Kellmann J-W (2001). Interactions between the tomato spotted wilt virus movement protein and plant proteins showing homologies to myosin, kinesin and DnaJ-like chaperones. Plant Physiol Biochem.

[pbio-0060007-b013] Boyko V, Ferralli J, Heinlein M (2000). Cell-to-cell movement of TMV RNA is temperature-dependent and corresponds to the association of movement protein with microtubules. Plant J.

[pbio-0060007-b014] Prokhnevsky AI, Peremyslov VV, Dolja VV (2005). Actin cytoskeleton is involved in targeting of a viral Hsp70 homolog to the cell periphery. J Virol.

[pbio-0060007-b015] Kragler F, Curin M, Trutnyeva K, Gansch A, Waigmann E (2003). MPB2C, a microtubule-associated plant protein binds to and interferes with cell-to-cell transport of tobacco mosaic virus movement protein. Plant Physiol.

[pbio-0060007-b016] Laporte C, Vetter G, Loudes AM, Robinson DG, Hillmer S (2003). Involvement of the secretory pathway and the cytoskeleton in intracellular targeting and tubule assembly of Grapevine fanleaf virus movement protein in tobacco BY-2 cells. Plant Cell.

[pbio-0060007-b017] Bayer EM, Bottrill AR, Walshaw J, Vigouroux M, Naldrett MJ (2006). Arabidopsis cell wall proteome defined using multidimensional protein identification technology. Proteomics.

[pbio-0060007-b018] Roberts IM, Boevink P, Roberts AG, Sauer N, Reichel C (2001). Dynamic changes in the frequency and architecture of plasmodesmata during the sink-source transition in tobacco leaves. Protoplasma.

[pbio-0060007-b019] Chen Z (2001). A superfamily of proteins with novel cysteine-rich repeats. Plant Physiol.

[pbio-0060007-b020] Du L, Chen Z (2000). Identification of genes encoding receptor-like protein kinases as possible targets of pathogen- and salicylic acid-induced WRKY DNA-binding proteins in Arabidopsis. Plant J.

[pbio-0060007-b021] Zamyatnin A, Solovyev AG, Bozhkov PV, Valkonen JPT, Morozov SY (2006). Assessment of the integral membrane topology in living cells. Plant J.

[pbio-0060007-b022] Liarzi O, Epel BL (2005). Development of a quantitative tool for measuring changes in the coefficient of conductivity of plasmodesmata induced by developmental, biotic and abiotic signals. Protoplasma.

[pbio-0060007-b023] Tagami Y, Watanabe Y (2006). Effects of brefeldin A on the localization of Tobamovirus movement protein and cell-to-cell movement of the virus. Virology.

[pbio-0060007-b024] Wright KM, Wood NT, Roberts AG, Chapman S, Boevink P (2006). Targeting of TMV movement protein to plasmodesmata requires the actin/ER network: evidence from FRAP. Traffic.

[pbio-0060007-b025] Nebenführ A, Ritzenthaler C, Robinson DG (2002). Brefeldin A: deciphering an enigmatic inhibitor of secretion. Plant Physiol.

[pbio-0060007-b026] Robinson DG, Herranz J, Bubeck R, Pepperkok R, Ritzenthaler C (2008). Membrane dynamics in the early secretory pathway. Crit Rev Plant Sci.

[pbio-0060007-b027] Ritzenthaler C, Nebenführ A, Movafeghi A, Stussi-Garaud C, Behnia L (2002). Reevaluation of the effects of brefeldin A on plant cells using tobacco Bright Yellow 2 cells expressing Golgi-targeted green fluorescent protein and COPI antisera. Plant Cell.

[pbio-0060007-b028] Phillipson BA, Pimpl P, daSilva LL, Crofts AJ, Taylor JP (2001). Secretory bulk flow of soluble proteins is efficient and COPII dependent. Plant Cell.

[pbio-0060007-b029] Griesbeck O, Baird GS, Campbell RE, Zacharias DA, Tsien RY (2001). Reducing the environmental sensitivity of yellow fluorescent protein. Mechanism and applications. J Biol Chem.

[pbio-0060007-b030] Doyle T, Botstein D (1996). Movement of yeast cortical actin cytoskeleton visualized in vivo. Proc Natl Acad Sci U S A.

[pbio-0060007-b031] Brandizzi F, Frangne N, Marc-Martin S, Hawes C, Neuhaus JM (2002). The destination for single-pass membrane proteins is influenced markedly by the length of the hydrophobic domain. Plant Cell.

[pbio-0060007-b032] Saint-Jore-Dupas C, Nebenführ A, Boulaflous A, Follet-Gueye ML, Plasson C (2006). Plant N-glycan processing enzymes employ different targeting mechanisms for their spatial arrangement along the secretory pathway. Plant Cell.

[pbio-0060007-b033] Bayer E, Thomas CL, Maule AJ (2004). Plasmodesmata in Arabidopsis thaliana suspension cells. Protoplasma.

[pbio-0060007-b034] Czernic P, Visser B, Sun W, Savoure A, Deslandes L (1999). Characterization of an Arabidopsis thaliana receptor-like protein kinase gene activated by oxidative stress and pathogen attack. Plant J.

[pbio-0060007-b035] Ohtake Y, Takahashi T, Komeda Y (2000). Salicylic acid induces the expression of a number of receptor-like kinase genes in Arabidopsis thaliana. Plant Cell Physiol.

[pbio-0060007-b036] Fujiki M, Kawakami S, Kim RW, Beachy RN (2006). Domains of tobacco mosaic virus movement protein essential for its membrane association. J Gen Virol.

[pbio-0060007-b037] Drakakaki G, Zabotina O, Delgado I, Robert S, Keegstra K (2006). Arabidopsis reversibly glycosylated polypeptides 1 and 2 are essential for pollen development. Plant Physiol.

[pbio-0060007-b038] Jürgens G (2004). Membrane trafficking in plants. Ann Rev Cell Dev Biol.

[pbio-0060007-b039] Rodriguez-Boulan E, Kreitzer G, Musch A (2005). Organization of vesicular trafficking in epithelia. Nat Rev Mol Cell Biol.

[pbio-0060007-b040] Karimi M, De Meyer B, Hilson P (2005). Modular cloning in plant cells. Trends Plant Sci.

[pbio-0060007-b041] Earley KW, Haag JR, Pontes O, Opper K, Juehne T (2006). Gateway-compatible vectors for plant functional genomics and proteomics. Plant J.

[pbio-0060007-b042] Tian GW, Mohanty A, Chary SN, Li S, Paap B (2004). High-throughput fluorescent tagging of full-length Arabidopsis gene products in planta. Plant Physiol.

[pbio-0060007-b043] Sanford JC, Smith FD, Russell JA (1993). Optimizing the biolistic process for different biological applications. Methods Enzymol.

[pbio-0060007-b044] Taylor NJ, Fauquet CM (2002). Microparticle bombardment as a tool in plant science and agricultural biotechnology. DNA Cell Biol.

[pbio-0060007-b045] Clough SJ, Bent AF (1998). Floral dip: a simplified method for Agrobacterium-mediated transformation of Arabidopsis thaliana. Plant J.

[pbio-0060007-b046] Takeuchi M, Ueda T, Sato K, Abe H, Nagata T (2000). A dominant negative mutant of Sar1 GTPase inhibits protein transport from the endoplasmic reticulum to the Golgi apparatus in tobacco and Arabidopsis cultured cells. Plant J.

